# Drought and heat reduce forest carbon uptake

**DOI:** 10.1038/s41467-023-41854-x

**Published:** 2023-10-06

**Authors:** Sebastian Wolf, Eugénie Paul-Limoges

**Affiliations:** 1 Department of Environmental Systems Science, Physics of Environmental Systems, ETH Zurich, Zurich, Switzerland; 2 Swiss Federal Institute for Forest, Snow and Landscape Research (WSL), Forest Dynamics, Birmensdorf, Switzerland

**Keywords:** Biogeochemistry, Climate sciences

## Abstract

Climate extremes threaten the land carbon sink and it is important to understand their impact in a changing climate. A recent study provides new insights on reduced forest carbon uptake during the severe 2022 drought and heatwave across Europe.

Climate extremes such as drought and heatwaves are detrimental to society (e.g. food production, human health and energy resources) and to the functioning of terrestrial ecosystems. While droughts have been occurring for centuries, Europe has recently experienced an elevated incidence of these extremes^1^, and projections show an increased prevalence with climate warming^2^. This is concerning because drought and heat threaten ecosystem carbon uptake, which currently mitigates increases in atmospheric CO_2_ concentrations by offsetting one-third of anthropogenic fossil fuel emissions^3^. Although the link between drought and reduced carbon uptake is well established, important questions remain regarding the impact of recurrent droughts, the strength of seasonal and regional compensation effects^4^, land-atmosphere feedbacks that can exacerbate heatwaves^5^, and forest management strategies in a changing climate^6^.

Van der Woude et al.^7^ have taken advantage of recently available, near real-time data to show that the severe (i.e. intense and prolonged) drought and heatwave in 2022 reduced forest carbon uptake at local, regional and continental scales across Europe. These results are important because the drought and heatwave in 2022 was a recurrent event following those of 2003, 2010, 2015, 2018, 2019 and 2020. Recurrent events enable key insights for lagged ecosystem responses (or ‘legacies’)^8^ and for shifting risks in a warming climate that impact the forest carbon sink such as tree mortality, vulnerability to insect outbreaks and fires, or shifts in species composition and forest structure^6^. During summer 2022, large areas of Europe experienced drought and heat that were among the strongest during the past 20 years. Large-scale droughts and heatwaves evolve from stationary, high pressure blocking patterns in atmospheric circulation, which inhibit cloud formation and precipitation, and increase available energy at the land surface^9^. Enhancing (i.e. positive) land-atmosphere feedbacks then further exacerbate extremely dry and hot conditions as water transpired by plants and evaporated from soils is reduced (see Fig. 1, blue arrows)^5^. Evaporative cooling thus becomes less efficient and more of the available energy heats the air (see Fig. 1, orange arrows).

**Fig. 1:**
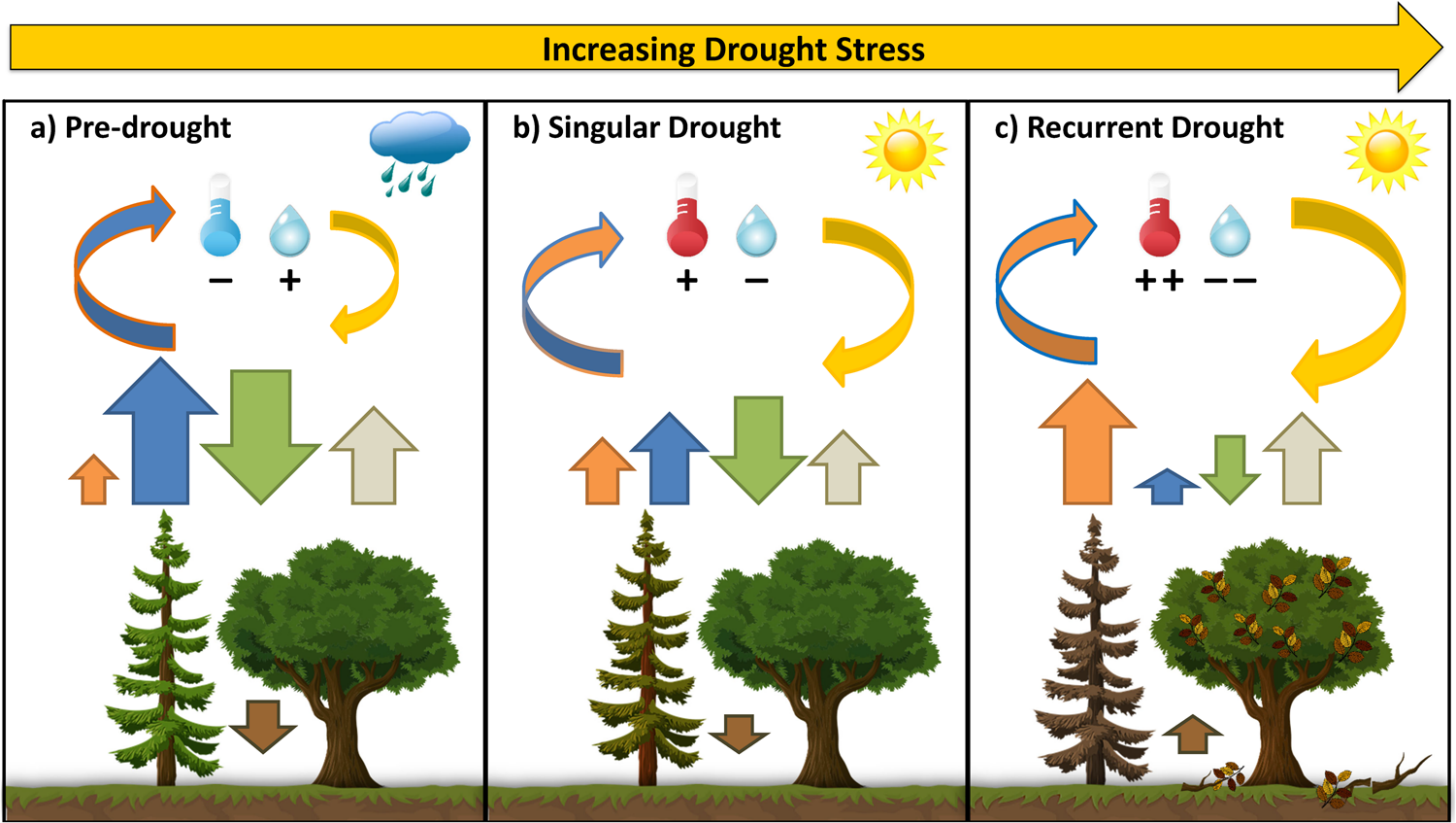
Impact of increasing drought stress on forest fluxes. Drought stress evolves (left to right) from a combination of precipitation deficits and heat. This leads to declines in soil moisture and increases in atmospheric evaporative water demand, which is a combination of air temperature and relative humidity (denoted by thermometer and water drop, respectively). Compared to pre-drought conditions (**a**), drought stress evolves during singular drought (**b**, i.e. meteorological and agricultural drought) and further increases during recurrent or prolonged drought (**c**, i.e. hydrological drought). Green arrows show carbon uptake (by photosynthesis), beige arrows carbon release (by soil and plant respiration), brown arrows the net carbon storage in forest or release to the atmosphere, blue arrows the water vapor flux (evapotranspiration), orange arrows the heat flux (sensible heat), and yellow arrows the drought stress. As drought stress increases, trees become physiologically stressed and reduce photosynthesis (denoted by change in tree color). After prolonged drought stress or recurrent drought, partial or full crown mortality occurs. Respiration is initially reduced by drought stress but eventually increases from decomposing leaves and wood. As drought stress increases, there is a shift from water vapor flux to more heat being released, enhancing the drought and heat conditions. (Source clip arts: Pixabay, modified)

How does drought and heat affect forest carbon uptake? The net carbon balance of an ecosystem represents the difference between carbon uptake by photosynthesis and carbon release by respiration. Reduced forest carbon uptake during drought and heat comes from stress-related declines in photosynthesis (see Fig. 1, green arrows). Respiration from plants and soil is also reduced due to limitations in soil moisture, but this is typically to a lower extent than photosynthesis (see Fig. 1, beige arrows)^10^. These relative differences result in reduced net carbon uptake (see Fig. 1, brown arrows) or even net release, as e.g. reported by van der Woude et al.^7^ for some sites in France during summer 2022.

The combined stress of intense drought and heat over prolonged periods (or recurrent events) leads to increased crown and eventually tree mortality (see Fig. 1c). Increased mortality has been reported for all major European tree species^11^, and the spatial distribution of these species regarding climatically suitable areas will be substantially altered by the end of this century^12^. For example, European beech is considered the most vulnerable broadleaf tree species to drought and heat^13^, and substantial climate-induced growth declines are projected^14^. This is important because beech forests cover vast areas of central and eastern Europe.

To quantify carbon uptake, van der Woude and colleagues^7^ combined ‘*bottom-up*’ ecosystem flux tower measurements in forests with ‘*top-down*’ approaches using satellite remote sensing observations and atmospheric inversions coupled with a biosphere model. Summer reductions of about 59 TgC were observed across the drought-affected area and resulted in 40 TgC reduced annual carbon uptake, which is equivalent to nearly one-quarter (23%) of the 2022 annual CO_2_ emissions of Germany – the European country with the largest emissions. Unlike in 2018 related to spring, only partial seasonal compensation was found from increased fall uptake due to a prolonged growing season. Similar carbon release from summer forest fires (about 9 TgC) make the 2022 event comparable with 2003, when much higher (up to 500 TgC) annual net carbon release was reported^10^. However, van der Woude et al.^7^ emphasize that other regions were affected in 2022 compared to 2018 and 2003, which likely mediated the impacts on reduced carbon uptake because of differences in forest composition.

While previous studies on the carbon cycle impact of climate extremes were delayed by data availability constraints^4,10,15^, van der Woude et al.^7^ demonstrate the importance of standardized ecosystem monitoring networks such as ICOS (European Integrated Carbon Observation System) and NEON (U.S. National Ecological Observatory Network) for providing timely information to stakeholders.

The severe impacts of drought on the forest carbon sink need to be considered by governments for reaching net-zero goals. Besides reducing deforestation, tropical countries like Brazil and Indonesia are planning substantial changes in land use (e.g. reforestation) to compensate for greenhouse gas emissions^16^. In Europe, forest area has already been increasing for decades (e.g. by 10% during 1990–2020) and land-use change (including reforestation) accounts for only 1% of the 2030 reduction target^16^. Essential in the European net-zero strategy is to retain the current forest carbon sink by adapting management practices, in particular because of the large uncertainty associated with climate change.

To ensure resilience of the forest carbon sink, improvements of current management practices (Fig. 2, *Forest box – Adaptation*) should include shifts to species mixtures (ideally of uneven age) that are better adapted to future climate conditions^12^, while preserving local species and biodiversity. Managed regeneration can prepare for species transitions following disturbances and enhance carbon uptake. In forests already resilient to drought and heat, the net-zero focus can shift towards increasing carbon storage to compensate emissions from other sectors (Fig. 2, *Forest box – Mitigation*), for example, through sustainable short-rotations, higher tree densities, or the introduction of more productive species.

**Fig. 2:**
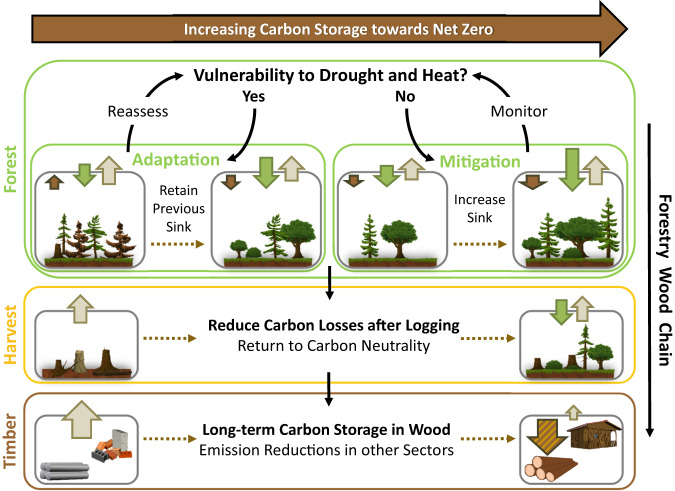
Forest management to increase carbon storage towards net zero, despite drought and heat. Management options for the forestry wood chain (Forest → Harvest → Timber) to increase carbon storage (left to right). Wide arrows show carbon uptake or release, i.e. forest uptake through photosynthesis (green arrows), release from respiration or industry to the atmosphere (beige arrows), and changes in net forest storage (brown arrows). The stripped brown arrow denotes long-term carbon storage in wood materials. Forest management strategies based on the vulnerability to drought and heat could retain the current carbon sink by adaptation efforts (if vulnerable) to avoid forest carbon release, or increase the sink by mitigation efforts (if not vulnerable) to compensate for emissions from other sectors. After wood harvest, carbon losses from soil and wood residues can be reduced to support a return to carbon neutrality. Storing carbon in wood materials increases stable long-term storage by reducing emissions from constructions materials such as cement and steel. (Source clip arts: Pixabay, modified)

For long-term carbon storage, wood materials arise as an interesting option because the storage effect is magnified by reducing large emissions from construction materials such as cement and steel^17^ (Fig. 2, *Timber box*). However, this option requires sustainable harvest management to minimize carbon release from deforested lands, such as by selective harvesting, supporting natural regeneration, and protecting soil and understory plants (Fig. 2, *Harvest box*).

Forest management strategies in a changing climate need to combine adaptation and mitigation approaches to enable resilient carbon storage, potentially complemented with long-term storage in wood products to further increase the mitigation potential. If implemented adequately, this could ensure compliance with the net-zero goal and make future forests more resilient against climate extremes like the 2022 drought and heat.

While the impacts of the 2022 event are still investigated, Europe is suffering yet another year with extreme temperatures and drought in 2023. Following a warm and dry winter, the Iberian Peninsula, southern France and northwestern Italy were affected by severe drought during late spring. Drought conditions then emerged in large parts of northern, central, and eastern Europe during June and July^18^, while record temperatures were observed along with a severe heatwave that peaked in late July^19^. The carbon cycle impact of this recent event remains to be determined, yet could provide further insights on legacy effects of recurrent drought and heat. The 2023 event might also rival the years 2018 and 2022 because of a combination of direct impacts (i.e. reductions in uptake, see Fig. 1) in the most affected regions, carbon release from widespread wildfires in southern Europe, and from accumulated legacy effects related to tree mortality from previous drought years.

The study by van der Woude et al.^7^ shows that the reduced forest carbon uptake during the 2022 drought and heat might be no longer exceptional in a warming climate, revealing the vulnerability of the forest carbon sink to such climate extremes. While it might be too early to designate such conditions the “*new normal*”, there is clear evidence that these events have been increasing in frequency and intensity across most of Europe^20^, and are projected to further increase with climate warming during spring and summer^2^. It is becoming apparent that recurrent drought and heat challenges the net-zero goals of governments relying on forestry, and that forest management needs to be adapted to retain the forest carbon sink.
